# Bioengineered microenvironment to culture early embryos

**DOI:** 10.1111/cpr.12754

**Published:** 2020-01-09

**Authors:** Zhen Gu, Jia Guo, Hongmei Wang, Yongqiang Wen, Qi Gu

**Affiliations:** ^1^ School of Chemistry and Biological Engineering University of Science and Technology Beijing Beijing China; ^2^ CAS Key Laboratory of Bio‐inspired Materials and Interfacial Science Technical Institute of Physics and Chemistry Chinese Academy of Sciences Beijing China; ^3^ State Key Laboratory of Membrane Biology Institute of Zoology Chinese Academy of Sciences Beijing China; ^4^ State Key Laboratory of Stem Cell and Reproductive Biology Institute of Zoology Chinese Academy of Sciences Beijing China; ^5^ University of Chinese Academy of Sciences Beijing China

**Keywords:** 3D bioengineer, biomaterials, embryo, microenvironment, stem cell

## Abstract

The abnormalities of early post‐implantation embryos can lead to early pregnancy loss and many other syndromes. However, it is hard to study embryos after implantation due to the limited accessibility. The success of embryo culture in vitro can avoid the challenges of embryonic development in vivo and provide a powerful research platform for research in developmental biology. The biophysical and chemical cues of the microenvironments impart significant spatiotemporal effects on embryonic development. Here, we summarize the main strategies which enable researchers to grow embryos outside of the body while overcoming the implantation barrier, highlight the roles of engineered microenvironments in regulating early embryonic development, and finally discuss the future challenges and new insights of early embryo culture.

## Graphical abstract

We summarize the regulation of the proliferation and differentiation of embryonic stem cells by adjusting bioengineered microenvironment, to make *ex vivo* culture of natural embryos and self‐assembly of embryonic stem cells mimic embryonic development *in vivo*, and future challenges are discussed finally.

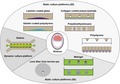

## 1 INTRODUCTION

Embryonic development is one of the most mysterious biological events.^1^, ^2^ Despite the extreme complexity of adult organisms, the entire growth of an embryo begins with the combination of an egg and a sperm. The seemingly bland monocyte is named as zygote.^3^, ^4^, ^5^ Under the right conditions, the fertilized egg will initialize a series of interacting and very precise genetic programmes and gradually develop into a completed living organism containing a series of tissues and organs which possess ordered patterns.^6^ There is always an unparalleled curiosity about embryonic development.^7^, ^8^, ^9^, ^10^ The early developmental processes of many species in the animal kingdom are surprisingly similar, with some genes or signals being slightly different. The molecular mechanisms underlying lineage specification before blastocyte stage in mice including humans has been unveiled as the pathways have been assumed to be conserved.^11^

After implanted in the uterus, the embryo progresses towards the crucial step gastrulation, which is hard to investigate. Therefore, the intervening period of development is still a big "black box".^12^ Fixed embryos at successive stages explain how the body is established, but more detailed information is unclear after the implantation. The ability to grow embryos from the blastocyst stage in vitro is particularly important to overcome this obstacle. The achievement of culturing post‐implantation embryos has enabled researchers to observe the embryonic morphogenesis and the occurrence of organs more intuitively. Besides, tracking those biological processes raises the possibility to better understand the complexities of development in the early stages, which may clarify why heart failure and other syndromes occur. Herein, we summarize the two major strategies of embryo culture in vitro: top‐down and bottom‐up, and the progress of research in the ex vivo embryo culture platforms. The main contents of this review include the background of natural embryos culture in vitro and the self‐assembly of embryonic stem cells (ESCs). Cultivation requires a more suitable static or dynamic culture platform, combined with monitoring embryo status in real time. Culture platforms, biomaterials, and various stimuli can be adjusted to mimic in vivo embryonic development. Finally, future research directions are discussed including key problems to be solved.

## 2 MAIN STRATEGIES OF THE EMBRYONIC DEVELOPMENT

Although the process of human embryonic development has been initially depicted in 1914,^13^ the embryonic development of the implantation stage has always been a "black box". The establishment of morphology and functionality during embryogenesis is an extremely complex process involving multiple levels of regulation.^1^, ^8^, ^14^, ^15^, ^16^ ESCs are laid out during the first few days of embryo implantation, with the overall morphological reorganization of embryos, the breaking of the symmetry of ESCs and the initiation of pedigree norms. The top‐down approach generally refers to the use of natural embryos as research objects for experimental manipulations and observations of the embryonic development.^17^ The study of embryonic development is challenging due to the small size and inaccessibility of the in vivo‐derived embryos. Inspired by synthetic biology, a simplified embryo model has been constructed using bottom‐up stem cell self‐assembly.^2^, ^15^, ^18^, ^19^ This pathway provides a simple system for studying early embryonic development which facilitates the experiment design visualization.^2^, ^17^, ^20^, ^21^, ^22^, ^23^

### 2.1 Top‐down

**Figure 1:**
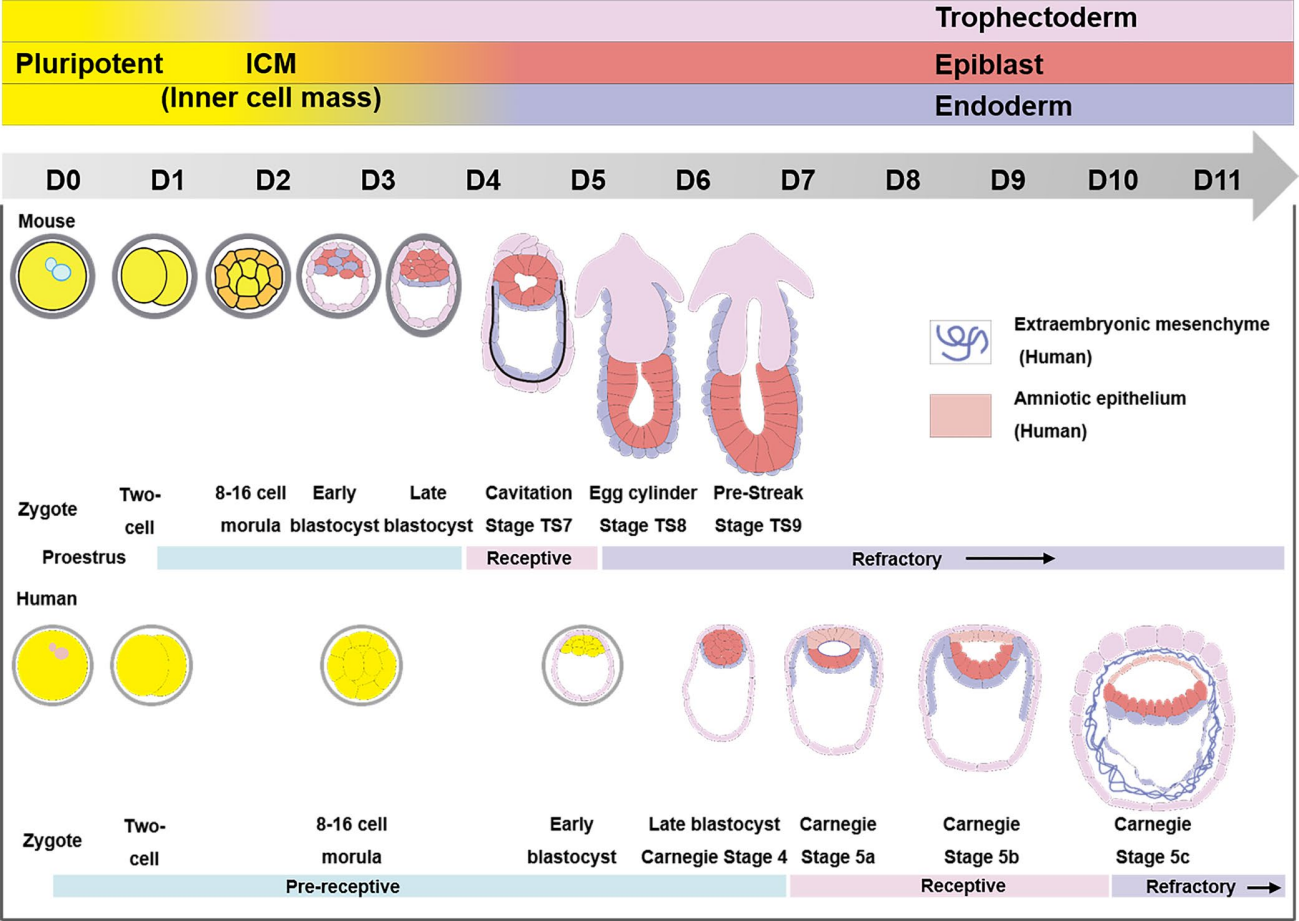
Mouse and human embryonic development and corresponding uterine status. The pre‐implantation development in mice and humans is similar, but with inconsistent periods.^29^, ^110^, ^111^ On D0, the sperms and oocytes combine to form a fertilized egg, and then the fertilized egg divides to form a multicellular aggregate. The 8‐cell late‐stage cells are divided into external cells and internal cells on D2 or D3. The external development to trophectoderm and the internal development to ICM happen on D3 or D4. Then ICM will develop into the extra‐embryonic cells and epiblast on D4 or D6. The TE part forms the epiplacental cone and the extraembryonic ectoderm on D5 or D10. The cells of the EPI part epithelialize, forming the anterior amniotic cavity on D6 or D10. Copyright 2017, Elsevier Inc^29^ and Copyright 2006, Nature Publishing Group^110^

One of the critical questions in life science is how embryo becomes a complex multicellular organism.^24^, ^25^ The top‐down approach generally refers to the use of natural embryos as research objects to evaluate embryonic development (Figure 1).^17^ In the early stage, mouse oocytes are fertilized and divided into the oviducts.^26^ With the increase of cell numbers, the embryo undergoes a compaction process at the 8‐cell stage. The blastomere is flattened, and the external cells are polarized, resulting in the internal and external differentiation at the stage of 8‐16 cells. The external cells are polarized to form trophectoderm (TE).^27^ Meanwhile, the first lineage is separated to form inner cell mass (ICM) and TE. ICM will develop into all tissues and organs of the foetus and some extraembryonic tissues while TE will develop into extraembryonic tissues.^28^ Cells continue to differentiate and then GATA4 or GATA6 positive primitive endoderm cells are found in ICM. The second lineage is separated to form primitive endoderm (PE) and epiblast (EPI) cells.^29^, ^30^ PE will develop into the extra‐embryonic yolk sac,^31^ where the blood cells of the embryo appear. EPI will develop into all tissues and organs of the foetus. The TE part forms the epiplacental cone and the ectodermal ectoderm. The cells of the EPI part are epithelialized to form a cavity. At the same time, the cells of the epidermal ectoderm are also epithelialized to form a cavity. Finally, the two walls are fused to form the pro‐amniotic cavity.^17^, ^32^ There are many similarities between the pre‐implantation developments in mice and humans (Figure 1).^29^ After the implantation in the uterus, the blastocysts move through the gut to form three germ layers.^10^, ^33^ The ectoderm will develop into the body's nerves, skin and other tissues. The mesoderm will develop into tissues such as the heart, blood, muscles and bones. The endoderm will develop into internal organs such as lungs, liver, pancreas and intestines.

### 2.2 Bottom‐up

The embryonic development is determined by multiple levels of the cell fate,^10^ forming the entire developmental blueprint of organogenesis and morphogenesis. Early embryonic development is accompanied by the maintenance of pluripotency, differentiation and the order of various pluripotent stem cells.^34^, ^35^, ^36^ However, natural embryos are small in size, bringing difficulties to conducting analytical studies, especially after the implantation. Embryoid bodies derived from in vitro cultured stem cells can help in understanding the specific history of the early embryonic development. Providing unlimited embryo supply and a new perspective on how embryos organize and grow, this pathway can be used for medical research and is expected to solve problems such as human infertility.^15^, ^18^, ^19^, ^20^, ^22^, ^37^

**Figure 2:**
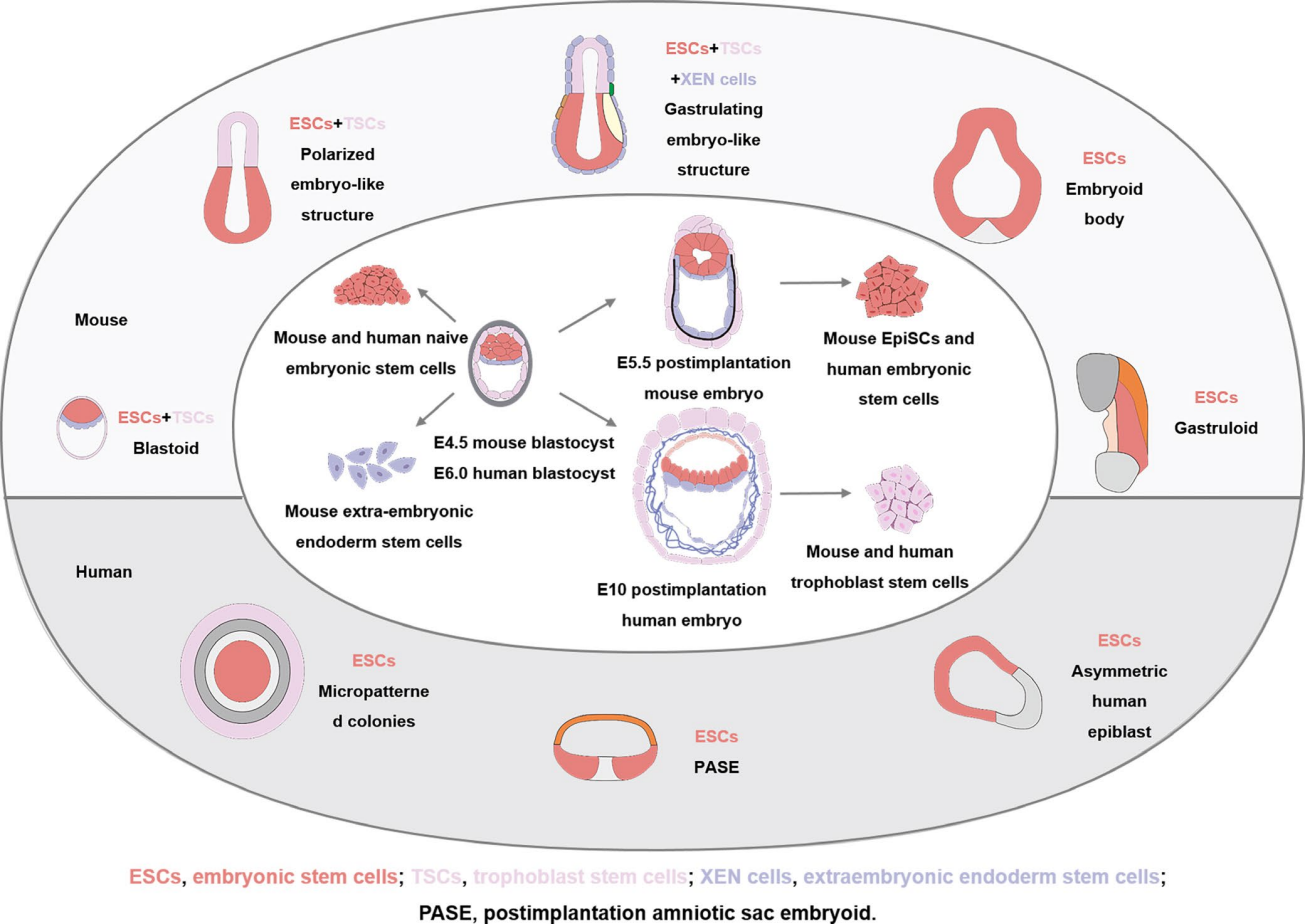
Mouse and human ESCs and their self‐assembling embryoid‐like structures. ESCs can form blastoid,^20^, ^40^ early post‐implantation,^41^ gastrulation^19^ and embryoid body.^112^ Copyright 2019, American Association for the Advancement of Science^2^

As early as 1985, it has been discovered that ESCs had the potential to mimic embryogenesis through forming embryoid structure, which is also the beginning of developing embryoid bodies.^38^ Currently, embryoid bodies are divided into mice and humans by species and classified at the cellular level into the following categories (Figure 2):^2^ ESCs, pluripotent stem cells (PSCs), extended pluripotent stem cells (EPS), ESCs + trophoblast stem cells (TSCs) and ESCs + TSCs + extraembryonic endoderm stem cells (XEN cells). The various stem cells play different roles in creating embryoid bodies. For example, embryos formed by ESCs can be used to explore the establishment of the three major axes during embryogenesis^39^; amnion‐like tissues are formed by PSCs^23^; embryoid bodies from EPS^40^ stimulate decidualization, and develop to cells of the three embryonic tissues; ESCs + TSCs compose embryo structures, which are similar to the morphology of natural embryos, and simulate the formation of blastocysts.^41^ Based on embryoid bodies from ESCs + TSCs, XEN cells are added and their position in the embryo is found close to where they are in natural embryos.^19^ The formation of embryoid bodies can be divided into four stages: blastocyst, early post‐implantation, gastrulation and post‐gastrulation. Blastocyst formation reveals that ES maintains TS proliferation and self‐renewal during embryogenesis and regulates trophoblast epithelial morphogenesis through BMP4/Nodal‐KLF6.^20^ Early post‐implantation illustrates the role of Nodal signalling during ETS‐embryogenesis.^41^ Gastrulation explains that WNT3A is not necessary for embryoid sac formation^7^; Post‐gastrulation reveals that during the formation of a mammalian gastrointestinal embryo, the physical stresses from cell‐matrix and cell‐cell are essential for the spatial self‐assembly of the germ layer.^42^

## 3 BIOENGINEERED MICROENVIRONMENT FOR EARLY EMBRYO CULTURE IN VITRO

In vitro embryonic development is closely related to the surrounding microenvironment.^1^, ^17^, ^43^, ^44^ To simulate the developmental processes of embryos in vivo,^45^ the spatiotemporal biophysical and biochemical microenvironments such as biomaterials, media and exerting forces^1^, ^46^, ^47^ are regulated to develop advanced bioengineered platforms for in vitro culture of embryos. It can be expected that the culture systems with continuous optimization, such as the establishment of a novel 3D culture system,^48^ will be more efficiently support embryonic development. Additional to this, various new technologies including gene editing^49^ and cytology analysis^8^ will surely help to unveil the mystery of the embryonic development gradually.

### 3.1 In vitro culture platforms for early embryo

Embryo culture in vitro is an effective method to study developmental biology. From the 1970s to the 1980s, there was an upsurge in the research of embryo culture in vitro*.* 50, 51, 52 However, no in‐depth molecular biological research was conducted due to the scientific and technological conditions at that time. Recently, with the development of sciences and technologies and the awareness of the importance of embryonic development after the implantation, researchers are returning to the study of embryonic development in vitro. Static culture platforms have been usually adopted for in vitro embryo cultures such as droplet culture,^53^ free‐floating culture^54^ and microwell culture.^20^ Static culture platforms are conducive to the accumulation of beneficial factors secreted by the embryo, but it also facilitates the enrichment of metabolic by‐products. Dynamic culture platforms can be used to continuously and uniformly provide a flowing medium including nutrition and remove metabolic by‐products. In addition to optimizing the in vitro embryo culture platforms, research effort is also put into the real‐time detection and characterization of embryos to achieve an automated and functionally integrated in vitro embryo culture platform.^55^

#### 3.1.1 Static culture platforms

**Figure 3:**
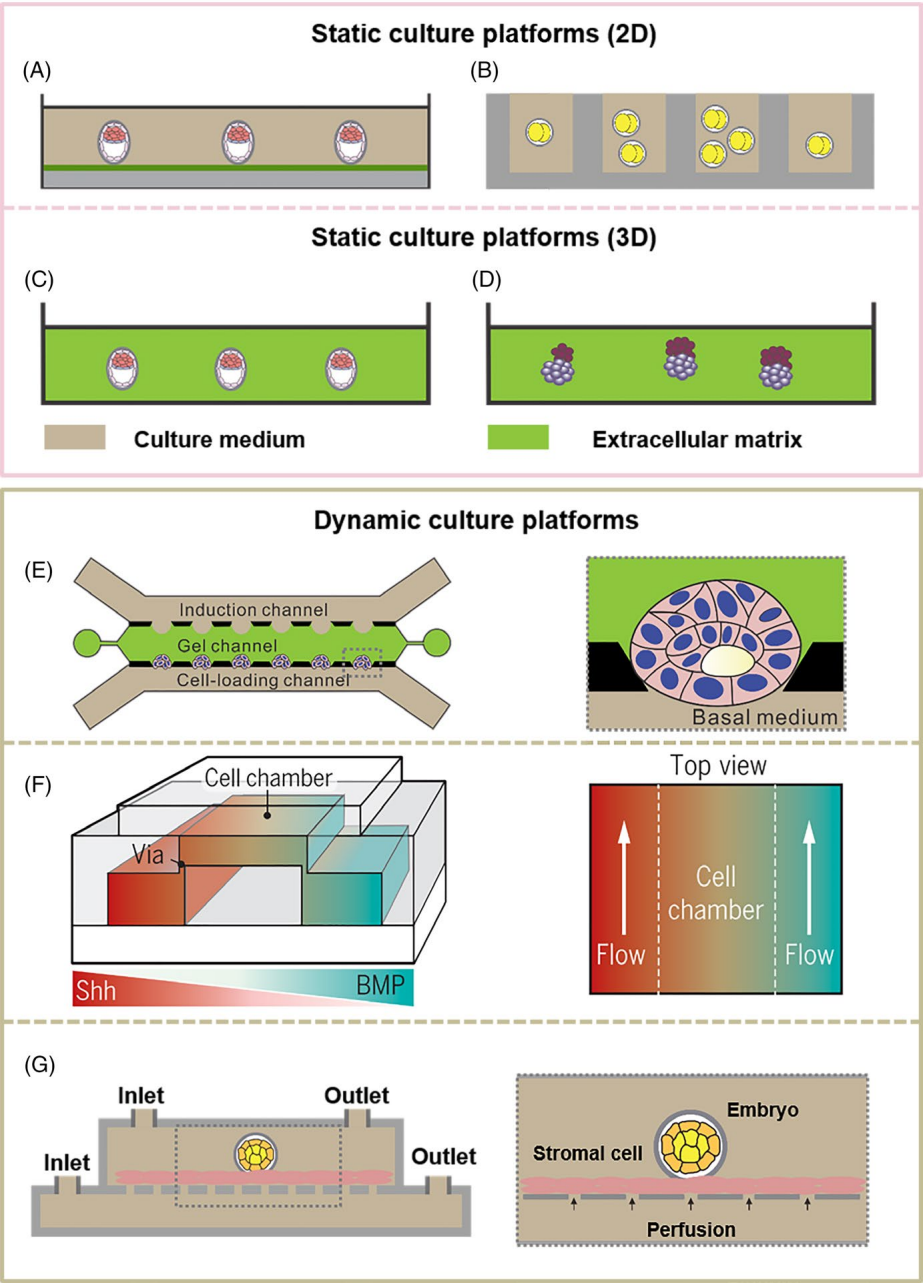
Typical platforms for embryo culture in vitro. (A‐D) Static culture platforms: (A) 2D in vitro embryo culture platform.^59^ Copyright 2014, Nature America, Inc^59^ (B) Microwell as in vitro embryo culture platform,^60^ which is commonly designed for single embryo culture and can improve contact between the zona pellucida and embryo. The number and size of the microwells and the volume of the medium may affect the culture outcomes. Copyright 2015, AIP Publishing LLC^60^ (C) 3D in vitro embryo culture platform.^58^ Copyright 2012, Kolahi et al^58^ (D) In 3D matrigel, mouse ESCs and extraembryonic TSCs self‐assemble to embryoid bodies.^41^ Copyright 2017, American Association for the Advancement of Science^41^ (E and F) Dynamic culture platforms: (E) An in vitro microfluidic culture system based on human pluripotent stem cells (PSCs).^7^ In this system, human PSCs can closely simulate the development of several key stages in the early implantation of the human embryo, which is also highly controllable and repeatable. Copyright 2019, Springer Nature^7^ (F) In a microfluidic system with a pair of channels,^66^ the gradient is generated by the diffusion of molecules (sonic hedgehog [Shh] and bone morphogenetic protein [BMP]), which helps to induce the formation of neural tube patterning. Copyright 2016, The Company of Biologists Ltd^66^ (G) Co‐culture platform of endometrial stromal cells and embryos.^67^, ^68^ Copyright 2013, Elsevier Limited^67^

Static culture platforms are usually fabricated from glass, plastic^54^, ^56^ or containers with the surface modified by an extracellular matrix^12^, ^57^ (Figures 3 and 5). The physicochemical microenvironment surrounding the embryo is generally considered to be constant. The static culture platforms are improved from the aspects of biomaterial modulus,^58^ light transmittance,^59^ medium volume^60^ and embryo density.^61^ Based on this, 3D embryo culture system has been developed to enable stem cells to self‐organize into embryo‐like structures without the external guidance.^41^

Bedzhov et al^59^ removed the zona pellucida from the early blastocysts which were subsequently cultured in a µ‐plate (Ibidi) (Figure 3A). Polyacrylamide hydrogels were cast on the µ‐plates and coated with collagen. After 5 days, the blastocyst developed to the egg cylinder stage. This culture platform has excellent light transmission and can be used to observe the embryos after the implantation in real time.^59^ Chung et al developed the microwells culture platform (Figure 3B), which was simple to prepare and could be applied to real‐time monitoring of embryonic development. The success rate from the two‐cell to the blastocyst stage reached 89%. After blastocysts were transplanted into the uterus of a mouse, they had the ability to develop to normal mice.^60^ Kolahi et al analysed the effects of biomaterial modulus on embryonic development before and after the implantation (Figure 3C). Compared with the traditional polystyrene culture dish, using collagen with modulus similar to that of biological tissues to culture embryos can increase the success rate of embryonic development and cell numbers before embryo implantation. These improvements will further increase the placental volume of the embryo during post‐implantation.^58^ Under 3D in vitro culture conditions, ESCs with self‐assembling properties can develop into natural embryos‐like structures.^18^, ^34^, ^42^, ^62^, ^63^ For example, Harrison et al^41^ found that in 3D matrigel, after 96 hours, ESCs can self‐assemble into a cylindrical structure similar to a natural mouse embryo (Figure 3D). The discovery provides a powerful platform for studying the physical and molecular mechanisms of embryonic development.

#### 3.1.2 Dynamic culture platforms

Taking into account the development of the embryo in vivo, the surrounding microenvironment of the embryo is dynamic, such as muscle movement, epithelial cilia movement and maternal respiration.^64^ Through introducing a flowing medium in a microfluidic system during the embryo culture, the microenvironment can be brought close to the biological microenvironment by controlling the fluid spatiotemporal distribution, co‐culture and so on.^7^, ^65^

The microfluidic system designed by Zheng et al^7^ includes three microfluidic channels (Figure 3E), which are used for embryonic‐like sac growth, PSC injection, and the delivery of morphogens required for cell differentiation. The system provides both a gel structure that mimics the wall of a uterus and nutrition continuously to these stem cells to facilitate their development. This system can successfully simulate the key processes of post‐implantation human embryos. It not only helps to enhance our understanding of the early development of human embryos but also bypasses the bioethical issues related to human embryo research. Signal molecules control the differentiation of PSCs during embryonic development with specific spatial and temporal distributions.^66^ Demers et al designed a dual‐channel microfluidic system (Figure 3F), which can provide ESCs in the central culture chamber with signal molecules with concentration gradients, simulating the natural spatiotemporal distribution, thereby inducing the formation of neural tube patterns. This provides an effective solution to the control of the microenvironment of embryo culture in vitro. Compared with traditional culture dishes, co‐culture of embryos and endometrial cells (Figure 3G), can improve the success rate and speed of embryonic development.^67^, ^68^

#### 3.1.3 Characterization of in vitro early embryo culture

**Figure 4:**
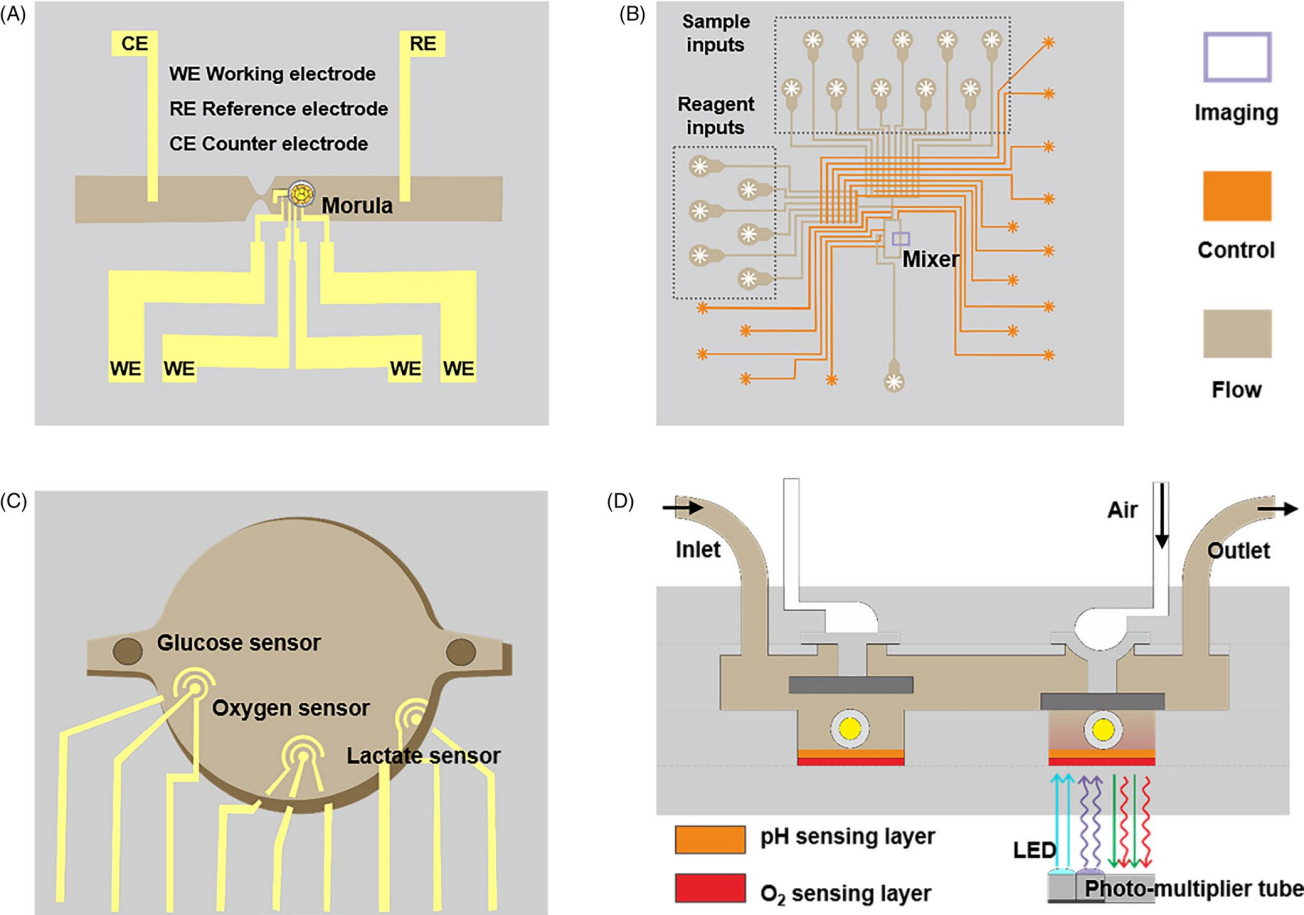
Real‐time monitoring of embryonic development in vitro. (A) A microchip for in situ monitoring the oxygen consumption of embryos.^69^ Copyright 2007, Elsevier BV^69^ (B) A microfluidic detector was applied to continuously evaluate embryo metabolising: glucose, pyruvate and lactic acid.^70^ Copyright 2008, American Chemical Society^70^ (C) Three sensors in the microchamber were designed to measure the concentrations of dissolved oxygen, glucose and lactic acid in embryo metabolism.^71^ Copyright 2019, Elsevier BV^71^ (D) A light modulated microfluidic device was developed for multiple long‐term measurements of oxygen consumption and acid extrusion in embryo metabolism.^72^ Copyright 2016, Springer‐Verlag Berlin Heidelberg^72^

To select well‐developed embryos, researchers have developed embryo characterization and evaluation platforms. The oxygen consumption, the utilization rate of glucose and other morphological parameters can be measured during in vitro embryo culture.^55^, ^69^, ^70^, ^71^, ^72^ To assess the viability of embryos, Wu et al^69^ designed a chip with a built‐in amperometric detector (Figure 4A), which can measure the oxygen consumption of a single embryo in situ. Urbanski et al^70^ developed a non‐invasive fluorescent detection platform (Figure 4B) that can detect embryo metabolites such as glucose, pyruvate and lactic acid with high sensitivity, high throughput, and automation. Obeidat et al designed a multi‐sensor microfluidic system (Figure 4C), by monitoring the metabolites of the embryos in real time. The glycolytic metabolic demand was enhanced when the embryos were developing from morula to blastocysts.^71^ This shows the potential of using the platform in the embryo metabolism research. Huang et al prepared a microfluidic device with a dual‐luminous sensor (Figure 4D), which can simultaneously monitor changes in oxygen consumption and pH values. The device is easy to manufacture and can facilitate high‐throughput monitoring.^72^

### 3.2 Microenvironment for in vitro early embryo culture

At present, the biomaterials required for in vitro embryo culture mainly include extracellular matrix materials and synthetic polymers, and their composites. The extracellular matrix materials have intrinsic biological activity,^73^ while the synthetic polymers perform excellent controllability.^74^ The combined use can take the advantages of both components. At the same time, in vitro microenvironments such as mechanics and topology also have important effects on embryonic development.^1^

#### 3.2.1 Biomaterials for in vitro early embryo culture

**Figure 5:**
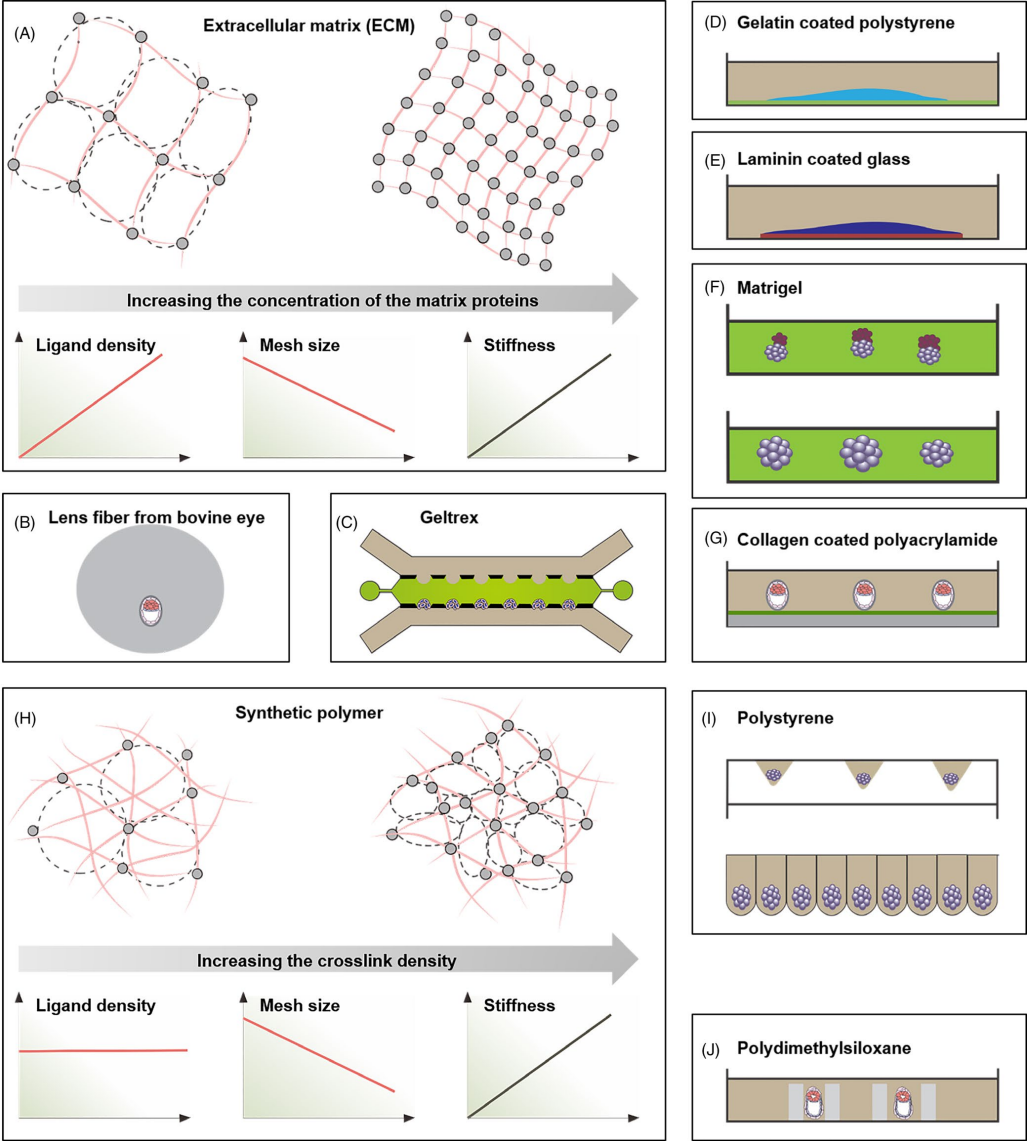
Biomaterials for early embryo culture in vitro. (A) As the concentration of extracellular matrix materials increases, the functional group density increases, the mesh size decreases and stiffness increases.^113^ Copyright 2017, Macmillan Publishers Limited^113^ (B) The earliest in vitro embryo culture is to place embryos in the transparent lens of a bull's eye.^50^ (C) The hollow spherical cavity is prepared by Geltrex.^7^ Copyright 2019, Springer Nature Limited^7^ (D and E) Mouse ESCs and human ESCs aggregates were cultured on gelatin‐coated polystyrene and laminin‐coated glass, respectively.^57^, ^77^ (F) The mouse ESCs and mouse TSCs (top) and mouse ESCs (bottom) are cultured using the matrigel embedding method.^41^, ^114^ (G) Mouse blastocysts are cultured on collagen‐coated polyacrylamide.^12^ (H) With the increase of crosslinking density, the ligand density of the synthetic polymer remains unchanged, the mesh size decreases, and the stiffness increases,^113^ Copyright 2017, Macmillan Publishers Limited^113^ (I) The mouse ESCs are cultured on a polystyrene substrate using a free‐floating method.^54^, ^56^, ^82^ (J) Mouse embryos are cultured in PDMS microcavities in different diameters.^115^ Copyright 2016, Springer‐Verlag Berlin Heidelberg^72^ and Copyright 2019, WILEY‐VCH Verlag GmbH & Co. KGaA, Weinheim^45^

The living individual, from cell level to the entire organism level, may have the ability to sense and respond to the characteristics of surrounding biomaterials.^74^, ^75^, ^76^ Embryo implantation requires direct interaction with the maternal and thus the biomaterials for culturing the embryo in vitro is essential. The current research mainly focuses on the source (extracellular matrix or a synthetic polymer) and the modulus of the biomaterials (Figure 5).

Current research continues to suggest that the nature of biomaterials affect the development of embryos (Figure 5), by providing mechanical support and physicochemical signals.^7^, ^57^, ^73^, ^77^ For example, Young's modulus of the culture plate used for embryo culture is about 1 GPa, which is 6 orders of magnitude of the uterine epithelium.^78^, ^79^, ^80^ It has been reported that biomaterials with Young's modulus similar to the uterine can effectively promote the development rate of embryos at 2‐cell stage and blastocyst stage.^58^, ^81^ Although synthetic polymers have been successfully used for free‐floating culture of mouse ESCs (Figure 5I),^54^, ^56^, ^82^ extracellular matrix materials have unique advantages in embryos culture in vitro due to their fine structures and complex biochemical characteristics.^73^ Besides, stem cells have begun to deposit extracellular matrix molecules during embryonic development.^83^ At present, embryos are mainly cultured in 2D and 3D microenvironments using extracellular matrix materials. To closely mimic the microenvironment in vivo, embryos are embedded in 3D extracellular matrix materials. The 3D microenvironment facilitates the self‐assembly of ESCs and TSCs into embryoid bodies (Figure 5F).^41^ However, there is no clear conclusion on which kind of biomaterial is optimal for the embryo culture. The cell‐biomaterial interface can be regulated through adjusting mechanical stiffness, wettability, electrical conductivity, magnetism, surface morphology and spatial structure.^84^, ^85^, ^86^, ^87^ Thereby the fate of cells can be manipulated, such as attachment, migration, proliferation, apoptosis, differentiation, etc.^88^, ^89^, ^90^, ^91^, ^92^, ^93^ Last but not least, there may be similar regulatory effects of different biomaterials on the embryonic development.

#### 3.2.2 Geometry and mechanics for in vitro early embryo culture

**Figure 6:**
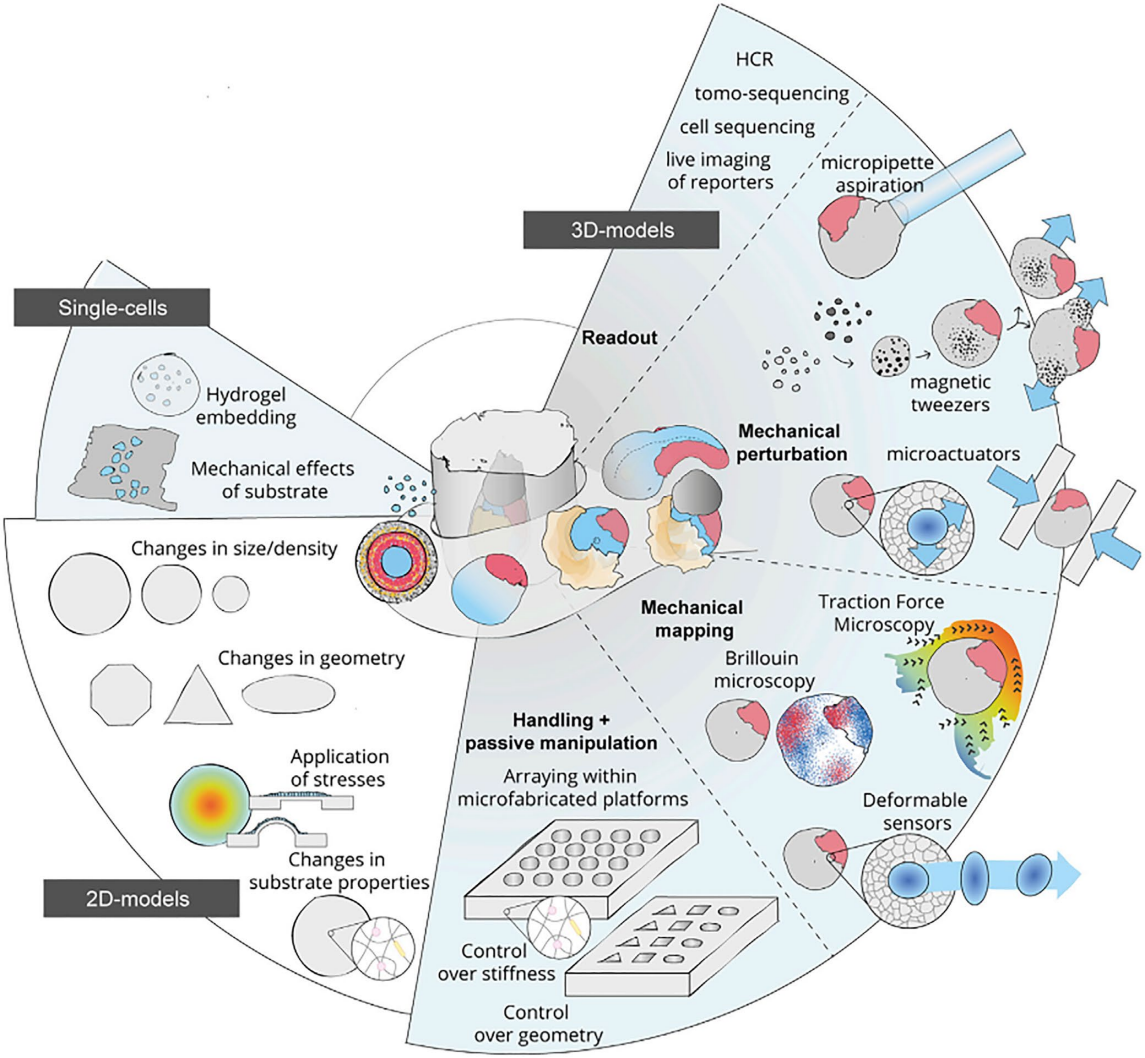
From a single cell to 3D models, embryonic development is regulated by mechanics and geometry.^1^ The mechanical properties such as the stiffness of the hydrogels around the cells can regulate the development of the cells.^94^, ^113^ Micropipette aspiration can be used to determine the tensions of cells in the embryo.^95^ Using 2D models, researchers can use micropatterning to control the self‐assembly of stem cells.^35^, ^57^, ^96^ Furthermore, the researchers applied stress to regulate embryonic spatial patterning.^47^, ^97^ Copyright 2019, Elsevier Inc^1^

The in vitro culture of the embryo overcomes the inconvenience of detecting development in vivo. As shown in Figure 6, the embryo's response to different external conditions can be studied, including external forces, topological structures, etc.^1^

From cellular levels to 3D models, both the microenvironment of mechanics and geometry play significant roles in embryonic development.^1^ The mechanical microenvironment has a great spatiotemporal influence on cell fate,^94^ which provides a design method for regulating cells. For instance, using micropipette aspiration to measure the tension of cells in the embryo can benefit in understanding the mechanism of the embryo compaction.^95^ Researchers use spatial geometric patterns to achieve the self‐assembly of ESCs. The cells are placed in a narrow circulation model of a special glass plate that can be chemically treated to form a microscopic pattern to prohibit the expansion of stem cells. When chemical signals are introduced into the narrow circulation model, they would stimulate stem cells to form gastrula. Stem cells can self‐assemble into endoderm, mesoderm and ectoderm tissues according to a certain geometric pattern under natural conditions.^35^, ^57^, ^96^ After the reported stress‐adjusted neuroectoderm developmental model,^47^ Chan et al^97^ utilize mouse blastocysts as a model to reveal the significant role of the fluid‐filled cavity in controlling embryo size and determining cell fate. During the development of the blastocyst, the pressure in the fluid‐filled cavity triples, and this increase leads to a simultaneous increase in cell cortical tension and tissue stiffness of the trophectoderm on the inner wall of the fluid‐filled cavity. Damaged tight junctions or increased tissue stiffness result in smaller embryo sizes.^97^

## 4 CONCLUSIONS

**Figure 7:**
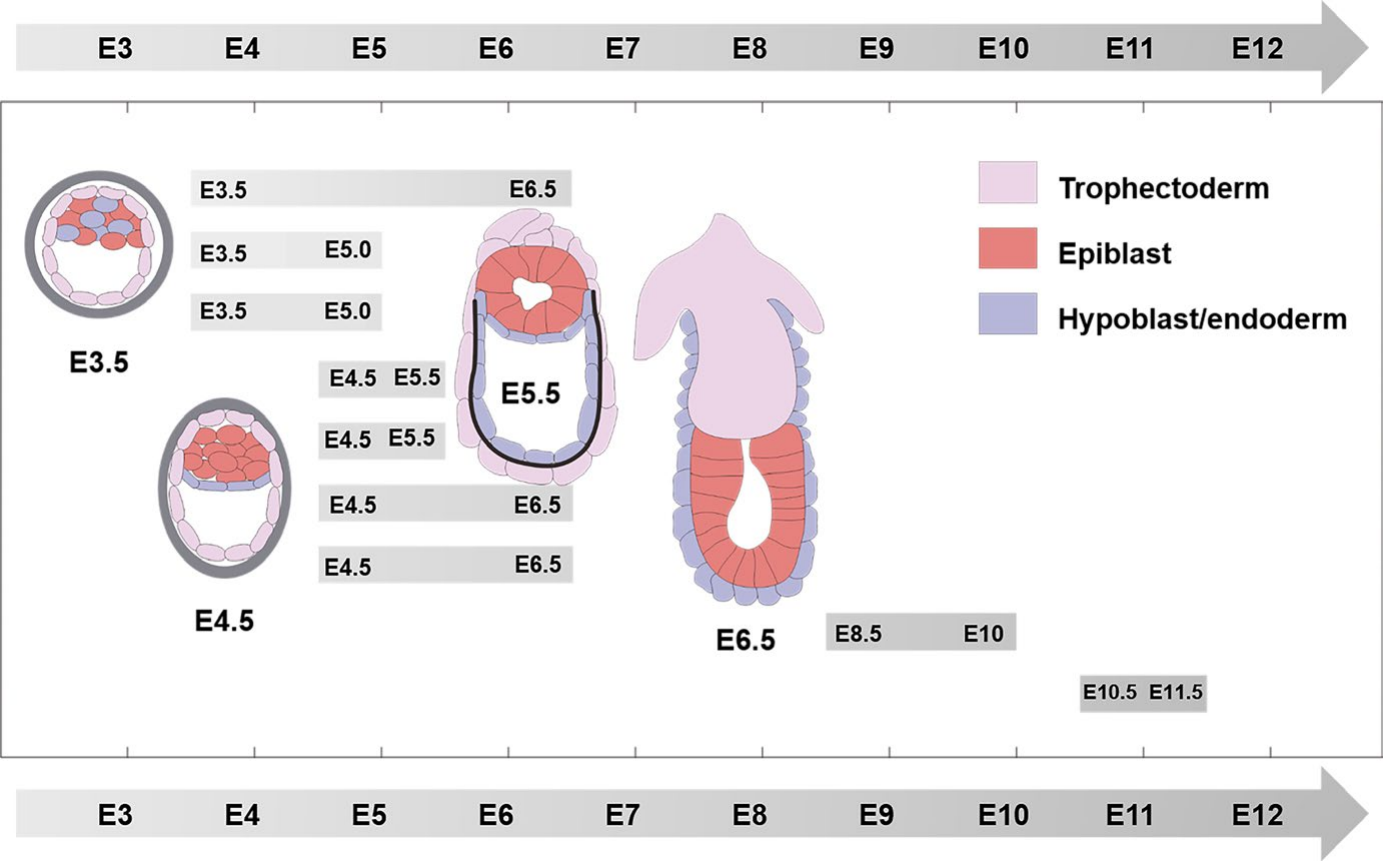
Mouse embryos are cultured in vitro, from different starting culture stages to various developmental stages, respectively. Embryos are cultured mainly from E3.5^12^, ^59^, ^116^ and E4.5,^41^, ^59^, ^115^, ^117^ and generally can be cultivated to E6.5. Starting the cultivation from a later stage, such as from E8.5 and E10.5,^118^, ^119^ is required to reach a longer cultivation state. Copyright 2017, Elsevier Inc^29^

This review summarizes the main strategies for in vitro culture of natural embryos and stem cell‐derived embryoid bodies by regulating the bioengineered microenvironments. Combined with in situ monitoring of embryonic development, the static and dynamic culture platforms are designed to closely mimic in vivo embryo microenvironments through exploring suitable biomaterials and external stimuli. However, ex vivo embryo culture is still particularly challenging. During the fused ES‐TS cells by fusing haploid ESCs and haploid TSCs are designed to develop into early embryos in vitro, the following challenges require particular attention: as the expression profiles of ES and TS genomes are not similar,^98^, ^99^, ^100^, ^101^ the gene expression in the fused ES‐TS cells is complex^102^, ^103^, ^104^ and haploid cells are ploidy unstable, so the fused haploid ES‐TS cells may obtain triploid and tetraploid cells, which cannot occur during early embryonic development. Due to the limited resources and considerable ethical and legal constraints with 14‐day rule after fertilization,^105^, ^106^ the existing methods of in vitro culture of human embryos are still at the exploratory stage and far from meeting the requirements of the clinical applications.^7^ The models of embryonic development obtained from animals cannot be directly applied to humans.^29^, ^107^, ^108^ Special attention needs to be paid to the impact of differences between models of animals and humans on the clinical applications. Compared with embryos developed in vivo, the current embryos cultured in vitro are limited to time (Figure 7), incomplete in structure and lack of extraembryonic tissue. Addition to the above mentioned limitations, in vitro cultured embryos cannot match the size and the number of cells of natural embryos.^17^, ^109^ Bioengineered microenvironments including biomaterials and stimuli are expected to be adjusted to better mimic the in vivo microenvironments for the optimal development of the embryo in vitro.

## CONFLICTS OF INTEREST

The authors declare no conflict of interest.

## AUTHOR CONTRIBUTIONS

ZG, YW and QG designed the review and made a retrieval strategy; ZG and JG drafted the review text; ZG and JG drafted the tables and figures; ZG, JG, HW, YW and QG contributed to revision and finalization of the manuscript.

## Data Availability

Research data are not shared.
